# Partial replacement of soybean with local alternative sources: effects on behavior, cecal microbiota, and intestinal histomorphometry of local chickens

**DOI:** 10.3389/fvets.2024.1463301

**Published:** 2024-11-13

**Authors:** Sezen Özkan, Veysel Bay, Muazzez Cömert Acar, Servet Yalcın

**Affiliations:** Department of Animal Science, Faculty of Agriculture, Ege University, İzmir, Türkiye

**Keywords:** agri-industrial by-products, black soldier fly larvae, local strain, microbiota, intestinal histomorphometry, behavior

## Abstract

Interest in partially replacing soybean meal in poultry diets with alternative protein sources such as agri-industrial by-products and black soldier fly (BSF, *Hermetia illucens*) has gained significant attention due to sustainability concerns. This study aimed to evaluate the effects of broiler diets in which soybean meal was partially substituted with agri-industrial by-products with or without BSF larvae meal, on the behavior, intestinal histomorphometry, and microbiome profile of a local broiler chicken strain. There were three dietary treatments. (1) A corn-soybean-based diet (Control), (2) a diet in which soybean was partly replaced (SPR) with local agri-industrial by-products, namely sunflower meal, brewers' dried grain, and wheat middlings, and (3) a diet in which BSF (5%) meal was added to SPR (SPR+BSF). Behavior was recorded on days 14, 35, and 49 at the pen level. On day 55, intestinal segments and cecal contents were collected from eight chickens per pen for histomorphometry and microbiome analysis. Dietary manipulations did not affect the behavior of broiler chickens (*P* > 0.05) suggesting that the experimental diets had no influence on behavior. A significant interaction between the intestinal segment and diets revealed that the SPR and SPR+BSF diets decreased duodenal villus height (VH) compared to the control diet (*P* < 0.05). However, this effect was not consistent across all of intestinal segments. Diet did not affect villus height to crypt depth ratio (VH/CD; *P* > 0.05), indicating no significant impact on the absorptive capacity of the digestive system. Firmicutes and Bacteroidetes were the dominant phyla in the cecal samples. *Colidextribacter* and *Oscillibacter* spp. were more abundant in chickens fed the SPR diet compared to those fed the control diet. The SPR+BSF diet resulted in higher abundance of *Rikenella* and *Colidextribacter* spp. compared to the control diet, while *Desulfovibrio, Ruminococcus torques* group, and *Lachnoclostridium* were more abundant in the ceca of birds fed the SPR diet than those fed SPR+BSF. In conclusion, replacement of soybean with agri-industrial by-products and BSF larvae meal could regulate the cecal microbiota composition without negatively affecting the behavior and intestinal histomorphometry of the local chickens.

## Introduction

Soybean is one of the most important protein sources in broiler diets. There is increasing interest in partially replacing soybean meal with locally available alternative protein sources due to rising soybean prices, dependence on international sources, and the growing trend toward sustainability in broiler production. Agri-industrial by-products, such as sunflower meal, legumes, rapeseed meal, citrus waste, grape pomace, and brewers' dried grain can be included in broiler diets (1–4). Insect larvae, considered as a sustainable and environmentally friendly alternative in broiler diets, may have the potential to partly replace soybean meal (5, 6). Several studies have demonstrated the beneficial effect of *Hermetia illucens* (black soldier fly, BSF) larvae meal as a protein source, partially or totally substituting the soybean meal, on the growth performance of meat type birds (7–9). In a recent study, Acar et al. (4) reported that partial replacement of soybean meal with local agri-industrial by-products such as sunflower meal, brewers dried grain, and wheat middlings did not affect growth performance, feed consumption, and feed conversion of broiler chickens from a local strain. However, the inclusion of 5% BSF larvae meal in broiler diets, including agri-industrial by-products to partially replace soybean meals, improved the growth rate of chickens during the starter phase (4).

It has been well-documented that there might be associations between the behavior of chickens and nutritional profile (10) and the sources of protein in the diets (11). van Krimpen et al. (11) reported that the inclusion of meat and bone meal into laying hen diets as animal protein sources resulted in behavioral changes, namely increased foraging and walking activity and floor pecking (11). BSF larvae have been usually provided as live (12) or dried larvae (13) in chickens as enrichment, resulting in increased activity which is considered a positive welfare outcome. Studies have shown that whole live or dry insect larvae provision affected the welfare of broilers by increasing foraging activity (12) and improving fear behavior and footpad health (14). A 5% inclusion of dry larvae meal to a diet including agricultural by-products showed no significant effect on welfare-related traits such as fear and footpad health (4). However, the effects of BSF larvae inclusion together with agri-industrial by-products into the diet have not been investigated.

Protein sources may affect intestinal development and the microbial community of the ceca of broilers (15), depending on protein digestibility (16) that may produce toxic compounds detrimental to chicken performance and gut health and (17). Intestinal morphology is one of the indicators of the gut health (18, 19). Long villi and a higher ratio between villi height (VH) and crypt depth (CD) are essential for efficient digestion and absorption (20). The studies on sunflower meal and brewers' dried grain on intestine development and microbial community are limited. Shorter villus and higher crypt dept in duodenum and jejunum were observed by increasing the inclusion of sunflower meal to the diet from 70 to 210 g/kg (21). Parpinelli et al. (22) found no effect of brewers' dried grain inclusion to diets up to 100 g/kg from 1 to 21 d on intestinal morphology. There are conflicting results on the effect of BSF larvae on villi and crypt development. Studies on the effect of BSF larvae on villi and crypt development indicated negative effects on gut morphology, including lower VH and increased CD, at a high inclusion level of 15% BSF defatted larvae meal into broiler diets, compared to lower inclusion levels of 5 and 10% (23). Cutrignelli et al. (24) observed both positive and negative effects of complete replacement of soybean with BSF larvae on the villi and crypt development in different intestinal segments of laying hens. They reported higher VH in the duodenum but lower VH in the jejunum and ileum together with a lowered VH/CD ratio in the ileum of laying hens. However, Biasato et al. (25) found no effect of BSF larvae meal up to 10% on intestinal morphology in piglets. These pioneering studies suggest that the level of BSF inclusion affects intestinal histomorphological responses, though this impact depends on both species and the inclusion level.

Intestinal health is also influenced by the microbiota. The chicken intestine is dominated by several microbial communities, mainly bacteria (26). The bacteria in the chicken intestine digest fiber and produce a series of metabolites, including short-chain fatty acids (SCFA) (27). Among SCFAs, acetate, propionate, and butyrate play an important role in intestinal health and energy metabolism and have a positive impact on the immune system (28). *Bacteroides, Bifidobacterium, Ruminococcus*, and *Clostridium* spp. are involved in forming their SCFA metabolites (28). *Bacteroides* spp. participate in acetate and propionate production, whereas Firmicutes are involved in butyrate formation (29). The ceca are the most densely populated microbiota section of the chicken intestine, with ~1,000 different species (30). In the chicken cecum, Bacteroidetes, Firmicutes, Proteobacteria, and Actinobacteria were the dominant phyla, while *Alistipes* spp., *Ruminococcaceae*, and *Faecalibacterium* spp. were the dominant genera (31, 32). Gut microbiota can be affected by diet. However, it was shown that partially replacing soybean meal with sunflower meal did not affect the cecal microbiota of broilers (33). The inclusion of BSF larvae fat may have the potential to alter the microbial community in chickens (34) and turkeys (35) by reducing harmful bacteria, thus promoting health compared to a control soybean-based diet. In contrast to these findings, it was also demonstrated that including up to 20% BSF larvae meal in broiler diets did not change cecal microbiota (36).

This study aimed to evaluate the effects of broiler diets, where soybean meal was partially replaced with agri-industrial by-products with or without BSF larvae inclusion on the behavior, intestinal histomorphometry, and microbiota of broiler chickens from a local line. We focused on a local slow-growing line because although the market for slow-growing broilers is currently smaller than that for fast-growing broilers, it has become more popular during the last decade. Indeed, Altmann et al. (37) concluded that BSF larvae meal (10% of the diet) would be a suitable protein source for slow-growing chickens without any significant effect on growth and welfare traits. Anadolu-T, a registered local broiler strain in Turkey, has been considered for small local growers. We used chicks from the dam line of Anadolu-T, which has a relatively slower growth rate (38). It was hypothesized that the diets in which soybean meal was partially substituted with agri-industrial by-products with or without BSF larvae meal inclusion may positively affect locomotor and comfort behavior, which might be associated with improved welfare and promote cecal microbiota and histomorphometry of the intestine.

## Materials and methods

This paper presents behavioral and microbiota data from a large-scale project (SUSTAvianFEED, No: 2015) funded by Prima, as part of a series of studies growth, blood biochemistry, welfare (4), and meat quality (39). The experimental procedures were approved by the Ege University Local Ethics Committee of the Agriculture Faculty (Approval No: 2022/02, 3-12-7316).

### Housing and diets

A detailed description of the experimental design, diets, and nutrient composition, including the amino acid and fatty acid profiles of agri-industrial by-products and BSF meal were provided in Acar et al. (4) and Yalçin et al. (39). In brief, a total of 252 day-old chickens from a local line (Anadolu-T pure dam line) were reared at 18-floor pens in an environmentally controlled experimental poultry house until 55 days of age. The chicks were randomly distributed into three dietary groups. (1) A corn-soybean-based diet (Control), (2) a diet in which soybean was partially replaced (SPR) with local agri-industrial by-products (resulting in an average 24.85% reduction in soybean meal amount compared to the control diet), including sunflower meal, brewers' dried grain, and wheat middlings, and (3) a diet in which dried BSF (5%) larvae meal was added to SPR (SPR+BSF), reducing soybean meal inclusion by an average of 42.2%. The ingredients and nutritional composition of the diets are given in Table 1. In this project, the target overall reduction in soybean meal inclusion (averaged across the starter, grower, and finisher phases) was set to above 20% for the SPR diet and 40% for the SPR+BSF diet, compared to the control. The inclusion levels of sunflower meal, brewers' dried grain, and wheat middlings in grower and finisher SPR and SPR+BSF diets were 6.30, 3.08, 3.08 and 8.00, 4.00, and 4.00%, respectively (Table 1). In the starter SPR diet, the inclusion levels for sunflower meal, brewers' dried grain, and wheat middlings were 3.58, 2.58, and 2.58%, respectively. Because of the difficulty in balancing metabolic energy, the inclusion levels of these ingredients were 0.05% higher in the SPR+BSF starter diet than in the SPR diet (3.63, 2.63, and 2.63% for sunflower meal, brewers' dried grain, and wheat middlings, respectively). These replacement levels allowed a reduction in the amount of soybean meal by 13.11, 24.37, 37.07% for the starter, grower, and finisher phases of SPR diets, respectively, as compared to the control ones (22.85% in average). In SPR+BSF diet, the level of reduction in soybean meal inclusion into the starter, grower and finisher phases of SPR+BSF diet were 26.88, 41.58, and 58.19%, respectively (42.22% in average). Nutrient composition, amino acids and fatty acids content of agri-industrial by-products and BSF larvae meal are summarized in Table 2. All diets were isocaloric and isonitrogenic and corresponded to NRC requirements (40) except with crude protein level which has been reduced in line with the sustainability approach as reported by Liu et al. (28) to apply the sustainability goal of SUSTAvianFEED project. The levels of alternative by-products in the diets were determined by taking into consideration the results from earlier studies (21, 41, 42). Each dietary treatment had 6 replicate floor pens (14 chicks/pen, 25 kg/m^2^). A 23L:1D lighting schedule was applied for the first 3 days, and lighting was gradually reduced to 18 h by day 7, maintaining this schedule until the end of the experiment. Standard brooding and growing period temperatures were applied during the experiment.

**Table 1:** Ingredients and nutrient composition of the experimental diets^a^ for starter (0–10 d), grower (11–25 d), and finisher (26–55 d) periods.

	**Control**			**SPR**			**SPR**+**BSF**		
	**Starter**	**Grower**	**Finisher**	**Starter**	**Grower**	**Finisher**	**Starter**	**Grower**	**Finisher**
Corn	45.28	51.24	57.34	39.18	44.44	47.44	41.3	46.54	49.64
Wheat	11.86	14.86	15.00	12.5.0	14.50	15.50	12.18	14.50	15.50
Soybean meal	34.33	27.90	23.20	29.8	21.10	14.6	25.10	16.30	9.70
Sunflower meal	-	-	-	3.58	6.30	8.00	3.63	6.30	8.00
Brewers dried grain	-	-	-	2.58	3.08	4.00	2.63	3.08	4.00
Wheat middling	-	-	-	2.58	3.08	4.00	2.63	3.08	4.00
BSF larvae	-	-	-	-	-	-	5.00	5.00	5.00
Sunflower oil	5.88	4.00	3.00	7.13	5.5.0	5.00	4.88	3.20	2.70
Limestone	0.50	0.30	0.20	0.50	0.30	0.20	0.50	0.30	0.20
Dicalcium phosphate	1.00	0.80	0.60	1.00	0.80	0.60	1.00	0.80	0.60
Vit+ min Premix^b^	0.25	0.25	0.25	0.25	0.25	0.25	0.25	0.25	0.25
Sodium chloride	0.20	0.20	0.20	0.20	0.20	0.20	0.20	0.20	0.20
Lysine (HCL—78%)	0.50	0.30	0.15	0.50	0.30	0.15	0.50	0.30	0.15
Methionine dl (99%)	0.10	0.05	0.01	0.1	0.05	0.01	0.10	0.05	0.01
Threonine	0.05	0.05	-	0.05	0.05	-	0.05	0.05	-
Enzyme^c^	0.05	0.05	0.05	0.05	0.05	0.05	0.05	0.05	0.05
**Analyzed nutrient composition**									
ME^d^, kcal/kg diet	2,984	2,923	2,904	2,992	2,921	2,904	2,991	2,919	2,903
Crude protein, %	20.78	18.68	17.00	20.74	18.65	17.05	20.78	18.64	17.04
Ether extract, %	8.49	6.63	5.79	9.41	8.11	7.52	9.38	7.72	7.52
Crude fiber, %	2.91	2.61	2.35	3.70	3.69	3.77	3.94	3.92	3.99
Calcium, %	1.08	1.04	1.02	1.08	1.03	0.99	1.13	1.08	1.05
Total phosphorus, %	0.50	0.44	0.38	0.55	0.52	0.47	0.55	0.52	0.48
**Calculated amino acid composition, g/100 g**									
Methionine	0.611	0.502	0.418	0.567	0.438	0.337	0.546	0.426	0.314
Lysine	1.113	0.847	0.644	1.129	0.871	0.678	1.130	0.871	0.677
Tryptophan	0.046	0.055	0.058	0.049	0.057	0.062	0.050	0.058	0.064
Histidine	0.244	0.248	0.248	0.248	0.253	0.256	0.255	0.260	0.263
Threonine	0.687	0.629	0.536	0.714	0.677	0.597	0.798	0.749	0.669
Valine	0.323	0.350	0.365	0.419	0.438	0.337	0.493	0.572	0.629
Isoleucine	0.513	0.482	0.455	0.590	0.614	0.625	0.594	0.616	0.626

^a^Diets: Control: corn–soybean-based diet; SPR: the soybean in the control diet was partially replaced with local feedstuffs; SPR + BSF: black soldier fly dried larvae were added to the SPR diet.

^b^Vitamin+mineral premix: Provided per 2.5 kg feed of diet. Vitamin A, 15,000,000 IU, Vitamin D3, 3,000,000 IU, vitamin E, 50,000 mg, Vitamin K3, 4,000 mg, vitamin B1, 3,000 mg, Vitamin B2, 6,000 mg, Niacinamid, 40,000 mg, Vitamin B6, 5,000 mg, Vitamin B12, 30 mg, Calcium-D-Pantothenate 15.000 mg, Biotin, 75 mg, Folic acid, 1,000 mg, Choline Chloride, 400,000 mg, Manganese 80.000 mg, Iron: 60.000 mg, Copper, 5,000 mg, Zinc, 60.000 mg, Iodine, 2.000 mg, Selenium 150 mg.

^c^Rovabio (50 gr) + Natuphos E (100 gr) BASF.

^d^ME, Metabolizable energy.

**Table 2:** Crude nutrients, total amino acids and fatty acid (based on dry matter) composition of agri-industrial by-products and BSF larvae meal used in the diets.

	**Sunflower meal**	**Brewers dried grain**	**Wheat middlings**	**BSF larvae meal**
**Nutrients compositions, %**				
Metabolizable energy, kcal/kg	2,108	1,565	1,837	5,381
Dry matter	90.46	90.53	88.20	95.52
Crude protein	41.78	28.61	17.53	42.62
Ether extract	1.65	2.68	4.34	42.54
Crude fiber	14.37	20.20	7.94	11.04
Neutral detergent fiber	37.57	74.68	32.59	17.56
Acid detergent fiber	29.88	28.97	10.78	13.36
Acid detergent insoluble nitrogen	-	-	-	0.74
Crude ash	6.92	4.11	5.98	6.29
Starch	-	2.71	17.87	-
Total sugar	8.18	2.07	-	1.79
**Amino acids, %**				
∑Essential amino acids	13.94	7.82	6.70	10.62
∑Non-essential amino acids	17.26	8.62	9.17	8.25
∑Amino acids	31.20	16.44	15.87	18.87
∑**Fatty acids, g/100 g lipid**				
∑Saturated	34.04	30.71	21.78	75.18
∑Monounsaturated	31.79	20.20	19.92	17.69
∑ Polyunsaturated	43.62	59.47	71.32	11.38

On d 55, eight chickens with equal numbers of each sex from each diet were randomly selected and sacrificed by cutting the jugular vein to obtain samples for histomorphometric evaluation of intestinal segments and cecal microbial flora.

### Behavioral observations

Three pens per dietary treatment were included in the behavioral observations. The ethogram used in the experiment is given in Table 3. Scan sampling was used to record the number of birds performing one of the feeding, drinking, walking-standing (locomotor), and sitting-lying (resting) behaviors in each pen (43). Scans were conducted once every hour during the 18 h photoperiod on d 14, 35, and 49. In addition to the behavioral categories given above, pecking (objects, equipment, or other chicks), preening, dustbathing, leg-wing stretching, and wing flapping were also recorded in a minute at each scan time point. Due to the rare occurrence of dustbathing and wing flapping behaviors during the observations, dustbathing, wing flapping, preening, and stretching behaviors were pooled and presented as comfort behavior as suggested in previous studies (13, 44). Behavioral recordings were made by two observers at the pen level. One of the observers recorded four basic behaviors (feeding, drinking, locomotor, and resting), while the second observer counted other behaviors in a minute whenever they were performed. Therefore, the same bird might have been recorded in different categories of behavior (44). The numbers of chickens performing one specific behavior were averaged per replicate pen per day and expressed as a percentage (%) of the total number of birds.

**Table 3:** Ethogram used in behavioral observations.

**Behavior**	**Definition**
Eating	Pecking at food in feeder
Drinking	Drinking from nipple or drip cup
Locomotor	Walking as taking more than a step or standing
Resting	Sitting or lying on the floor
Pecking	Pecks on inedible objects including litter, environment, and pen mates
**Comfort behaviors**	
Dustbathing	Wing movements within contact to litter substrate with fluffed feathers and scratching litter
Preening	Grooming of feathers with beak
Wing flapping	Vertical wing shakes, sometimes running at the same time without stimulus
Stretching	Stretching of a leg and/or wing

### Intestinal histomorphometry

About 2 cm of samples from the duodenum (middle part), jejunum, and ileum (both one cm away from Meckel's diverticulum) were collected for intestinal histomorphometry measurements. The intestinal segment samples were rinsed with saline, fixed in 10% formalin solution, and maintained in the formalin at room temperature until analysis. The samples were washed, dehydrated with alcohol, and cleared with xylene before paraffin wax. Tissue samples were cut by a microtome and stained using hematoxylin and eosin. Five villi and crypt were randomly selected under the light microscope, and measurements of VH, villus width (VW), and CD were performed using the Sigma Scan Pro5 program (Systat Software, Inc, CA, USA). The ratio VH to CD was calculated (45).

### Sampling for microbiota analysis

Cecal content samples from chickens used for intestinal histomorphometric evaluations were collected into sterile tubes, placed on dry ice, and stored at −80°C until microbiota analysis.

### DNA extraction

DNA was extracted utilizing the QIAamp DNA Stool Mini Kit (Qiagen, Germany) according to the manufacturer's protocol. The concentration (ng/μL) and purity (A260/A280 and A260/A230 ratios) of the DNA samples were measured using a Nanodrop 8000c Spectrophotometer (Thermo Fisher Scientific, USA).

### 16S rRNA gene amplicon sequencing

Amplicon sequencing analysis was performed with eight chicken samples from each diet. PCR amplification of the targeted V3-V4 region was performed by using specific primers 341F (5′-CCTAYGGGRBGCASCAG-3′) and 806R (5′-GGACTACNNGGGTATCTAAT-3′). The Nextera XT DNA Library Preparation Kit (Illumina, USA) was utilized to generate sequencing libraries after the quantification and qualification of PCR products. The concentration of the libraries was normalized by diluting to 4 nM then libraries were sequenced on a paired-end Illumina NovaSeq 6000 platform to generate 250 bp paired-end (2 × 250 bp) raw reads. FastQC and QIIME2 were used to assess the raw data quality and read quality control, respectively. Effective tags were obtained by using DADA2 to remove primer and barcode sequences, chimeric reads, and reads with a Phred Score of < 20, hence improving the accuracy and reliability of the results. QIIME2 was used for the taxonomic determination of each Operational Taxonomic Units (OTUs) representative sequence. OTUs were annotated to obtain the corresponding species information and the abundance distribution based on the species with ≥97% similarity against the SILVA (138.1) (46). According to the results annotations of each sample, the species abundance tables at the level of kingdom, phyla, class, order, family, genus, and species were obtained. Since these abundance tables with annotation information were the core content of amplicon analysis, determination of relative abundance, and alpha and beta diversity analyses were carried out by selecting requested classification levels (e.g., phylum, genus). To clarify the richness and diversity of microbial communities in each sample, alpha diversity analyses were conducted. By using dimensionality reduction methods like PCoA in beta diversity analysis, the variations among several groups were investigated.

### Statistical analysis

Behavioral data were analyzed using the general linear mixed model procedure of JMP software (Pro-13). The model included diet and age as fixed effects and their interaction with a random effect of the pen. Before the statistical analysis, Shapiro-Wilk's test was used to ensure that the normality assumption of data was met. Shapiro Wilk's test confirmed normality assumptions for feeding, locomotor, resting, and comfort behavior. Because the drinking and pecking data did not follow a normal distribution, logarithmic transformation was applied before the analysis. However, actual values were presented in the tables. When a fixed effect was found to be significant, least square means were separated with Tukey test using JMP software. *P* < 0.05 was considered significant. The statistical model for histomorphometric measurements included diet and intestine part and their interaction. The significance of variations in bacteria composition and community structure of groups was tested using the *T*-test, Kruskal-Wallis, Anosim, and multiple response permutation process (MRPP) statistical tests. *P*-values below 0.05 were considered statistically significant. All statistical analyses were performed with R software (Version 4.3.1; https://www.r-project.org).

## Results

### Behavior

Table 4 presents the effect of diet and age on home-pen behavior of Anadolu-T chickens. There was no effect of diets and diet × age interaction on the percentage of birds in any behavioral category (*P* > 0.05). The age of chickens had a significant effect on feeding, locomotor, resting, pecking, and comfort behaviors (*P* < 0.05). The percentage of chickens performing feeding, locomotor, pecking, and comfort behaviors significantly decreased with the increasing age, while resting behavior increased in chickens with the increase of age (*P* < 0.05). There was no effect of age on the percentage of birds displaying drinking behavior (*P* > 0.05).

**Table 4:** The effect of diet and age on least square means for the percentage of chickens performing different behaviors.

	**Feeding**	**Drinking**	**Locomotor**	**Resting**	**Pecking**	**Comfort**
**%**						
**Diet** ^d^						
Control	27.38	5.92	14.12	52.58	15.89	34.10
SPR	24.89	6.55	17.49	51.07	15.57	32.43
SPR+BSF	25.88	7.02	18.55	48.55	16.02	35.15
SE^e^	1.833	0.914	1.909	3.157	0.596	1.269
**Age**						
14 d	30.68^a^	6.48	22.80^a^	40.04^b^	22.44^a^	39.81^a^
35 d	26.36^ab^	5.81	13.25^b^	54.58^a^	13.59^b^	33.81^b^
49 d	21.11^c^	7.20	14.11^b^	57.59^a^	11.44^b^	28.06^c^
SE	1.833	0.914	1.909	3.157	0.596	1.269
**Variation sources**	**Significance of** ***P*****-values**					
Diet	0.5961	0.8781	0.2905	0.6139	0.8828	0.4527
Age	0.0043	0.8089	0.0066	0.0011	< 0.0001	0.0002
Diet × Age	0.9228	0.6680	0.9055	0.9805	0.8593	0.8944
Pen	0.7734	0.0068	0.7545	0.7037	0.3995	0.0239

^a, b, c^Different superscript letters in columns indicate significant difference between the least square means (P < 0.05).

^d^Diet: Control: corn–soybean-based diet; SPR: the soybean in the control diet was partially replaced with local feedstuffs; SPR + BSF: black soldier fly dried larvae were added to the SPR diet.

^e^SE, Standard error.

### Intestinal histomorphometry

Shorter VH and lower CD were obtained in the ileum compared to the duodenum and jejunum (*P* < 0.05; Table 5). The duodenum had the largest VW compared to the jejunum and ileum (*P* < 0.05). Significant interactions were observed for histomorphometric measurements except VH/CD (*P* < 0.05). Chickens that consumed the SPR and SPR+BSF diets had a decreased VH in the duodenum compared to chickens fed the control diet (*P* < 0.05). The VH in the jejunum was shorter in chickens fed the SPR diet compared to those fed control and SPR+BSF diets (*P* < 0.05). The VH increased by the SPR diet compared to the Control and SPR+BSF diets in the ileum (*P* < 0.05). The SPR and SPR+BSF diets resulted in a narrow villus in all intestinal segments. The SPR and SPR+BSF diets did not influence CD except duodenum, where SPR+BSF diet resulted in a shorter CD (Table 5). The diet did not affect VH/CD ratio (*P* > 0.05).

**Table 5:** Effects of diets^d^ on villus height (VH), villus width (VW), crypt depth (CD), and villus-to-crypt ratio (VH/CD) of intestinal segments.

		**VH**	**VW**	**CD**	**VH/CD**
Intestinal segment (IS)		< 0.001	< 0.001	< 0.001	< 0.001
	Duodenum (D)	0.630^a^	0.0693^a^	0.0968^a^	7.075^a^
	Jejunum (J)	0.630^a^	0.0620^b^	0.0969^a^	6.988^a^
	Ileum (I)	0.408^b^	0.0606^b^	0.0840^b^	5.108^b^
	SEM^e^	0.008	0.0011	0.0020	0.178
Diet (D)		< 0.001	< 0.001	0.042	0.178
	Control	0.573^a^	0.0719^a^	0.0935^ab^	6.439
	SPR	0.539^b^	0.0613^b^	0.0954^a^	6.144
	SPR+BSF	0.555^ab^	0.0588^b^	0.0887^b^	6.588
	SEM	0.008	0.0011	0.0019	0.170
ISxD		0.008	< 0.001	0.0063	0.108
	D-Control	0.673^a^	0.0810^a^	0.1032^a^	7.124
	D-SPR	0.611^b^	0.0690^b^	0.1019^a^	6.632
	D-SPR+BSF	0.605^b^	0.0578^c^	0.0854^b^	7.470
	J-Control	0.648^a^	0.0667^a^	0.0955^a^	7.075
	J-SPR	0.563^b^	0.0607^b^	0.0958^a^	6.468
	J-SPR+BSF	0.679^a^	0.0589^b^	0.0992^a^	7.420
	I-Control	0.398^b^	0.0681^a^	0.0820^a^	5.120
	I-SPR	0.444^a^	0.0542^b^	0.0886^a^	5.331
	I-SPR+BSF	0.382^b^	0.0596^b^	0.0815^a^	4.873
	SEM	0.013	0.0020	0.0035	0.277

^a, b, c^The means in the same column with different superscripts differ significantly (p < 0.05).

^d^Diets: control: corn–soybean-based diet; SPR: the soybean in the control diet was partially replaced with local feedstuffs; SPR + BSF: black soldier fly dried larvae were added to the SPR diet.

^e^SEM, standard error of means.

### Microbiome composition of the cecum

**Figure 1:**
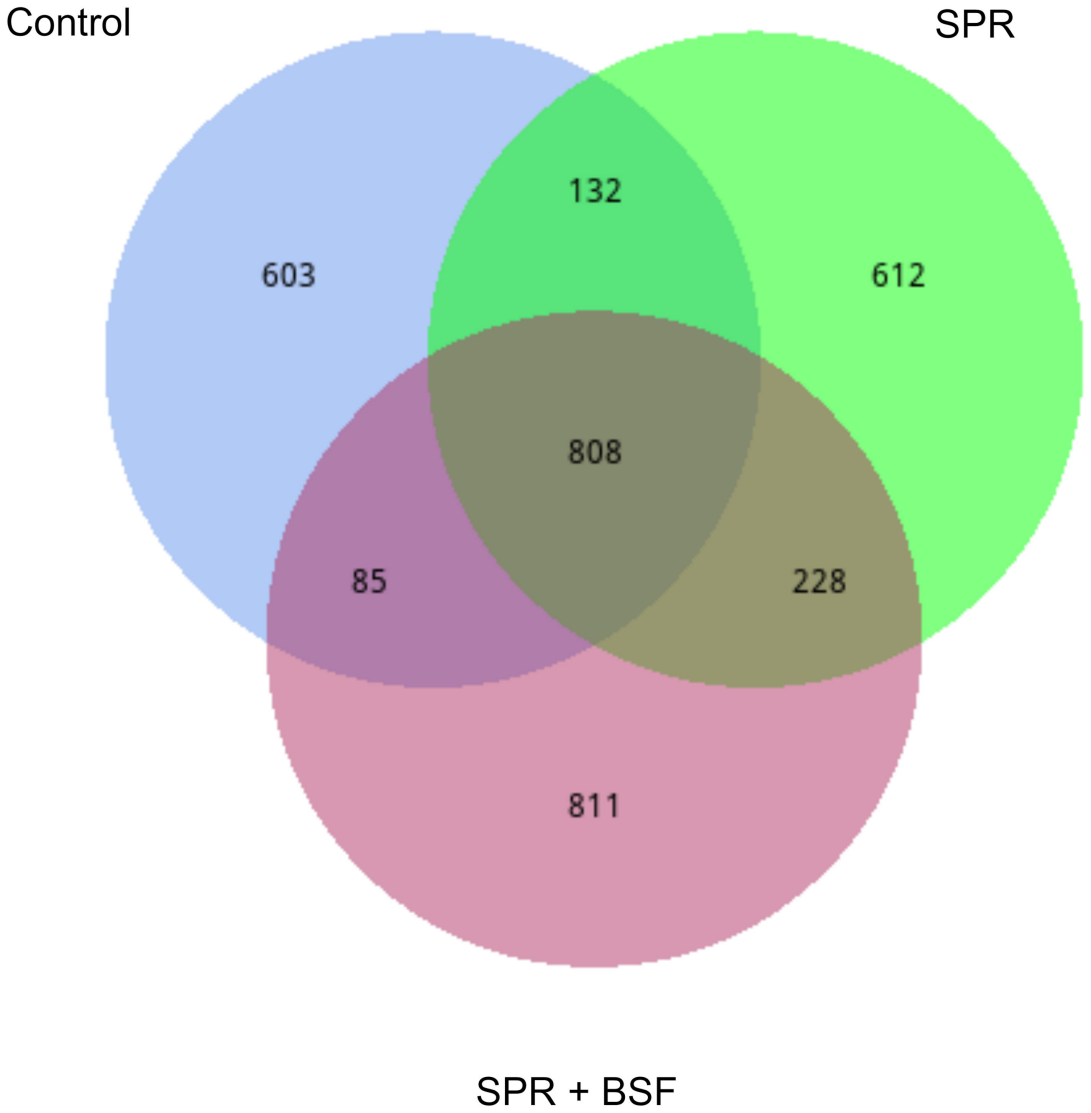
Venn diagrams illustrating the shared and unshared bacteria in the cecal samples of chickens fed different diets. Diets: control: corn–soybean-based diet; SPR: the soybean in the control diet was partially replaced with local feedstuffs; SPR + BSF: black soldier fly dried larvae were added to the SPR diet.

Venn diagrams illustrating the shared and unshared bacteria among the cecal samples of chickens fed different diets are given in Figure 1. There was a total of 808 shared Operational Taxonomic Units (OTUs). The ceca of chickens fed Control and SPR diets had 132 shared OTUs, while SPR and SPR+BSF had 228 shared OTUs. There were 603, 612, and 811 unshared OTUs in chickens fed Control, SPR, and SPR+BSF diets, respectively.

The major bacterial community characteristics are shown in Table 6. The richness of the bacterial community (Chao 1) in cecal samples was higher in chickens fed the SPR+BSF diet compared to those fed the Control diet, with the SPR diet showing intermediate values. No significant differences were found among cecal samples of chickens from different dietary groups for Shannon, Pielou's evenness, and Simpson indexes.

**Table 6:** Alpha diversity characteristics of cecal samples of chickens fed different diets^c^.

	**Alpha diversity indices**			
	**Chao1**	**Pielou_E**	**Shannon**	**Simpson**
Control	1,662.194^b^	0.668^a^	7.124^a^	0.970^a^
SPR	1,865.369^ab^	0.703^a^	7.588^a^	0.981^a^
SPR+BSF	1,998.828^a^	0.680^a^	7.423^a^	0.978^a^

n = 8.

^a, b^Means with the different superscripts within a column show significant differences (p < 0.05).

^c^Diets: control: corn–soybean-based diet; SPR: the soybean in the control diet was partially replaced with local feedstuffs; SPR + BSF: black soldier fly dried larvae were added to the SPR diet.

**Figure 2:**
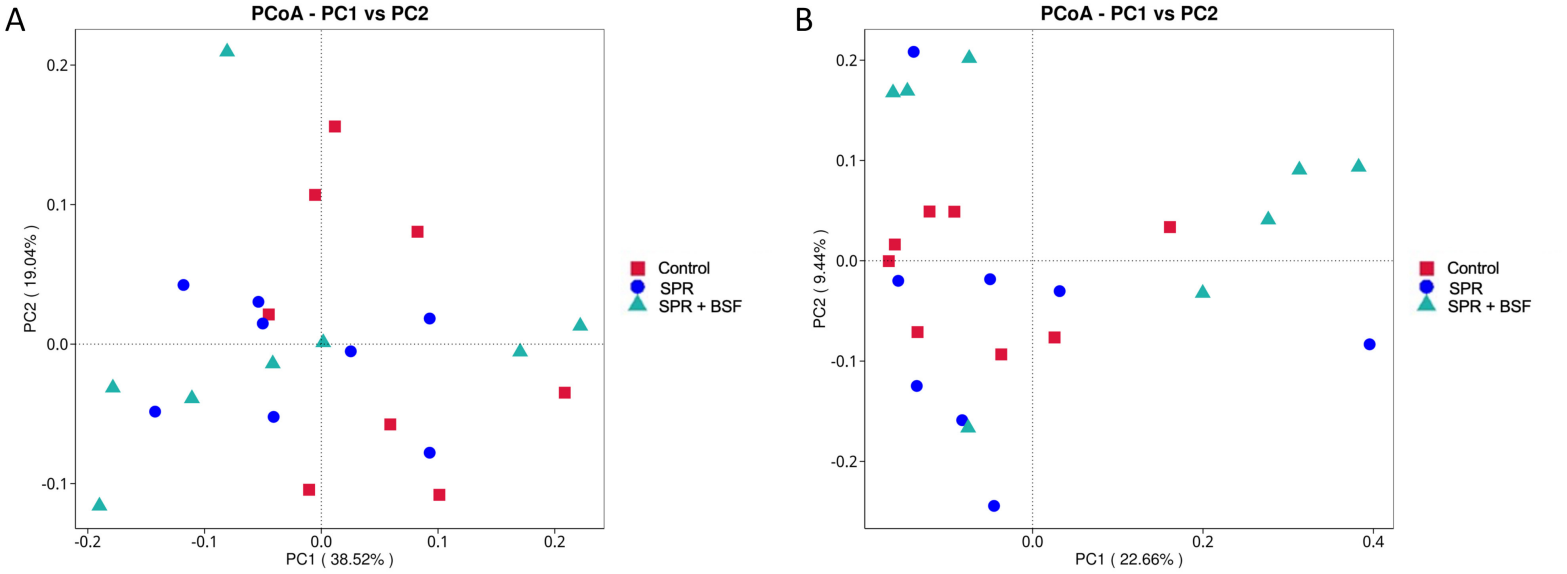
Unweighted and weighted UniFrac PCoA plot of cecal [**(A, B)**, respectively] microbiome composition of local chickens fed different diets. Diets: control: corn–soybean-based diet; SPR: the soybean in the control diet was partially replaced with local feedstuffs; SPR + BSF: black soldier fly dried larvae were added to the SPR diet.

PCoA was performed to evaluate the structural difference between the microbiota of different sample groups. The PCoA, based on the UniFrac distance, including unweighted and weighted values in the cecal samples of local chickens, is presented in Figure 2. The dietary groups showed no obvious differences in the composition of cecal microbiota in the PCoA distribution (Figures 2A, B).

**Figure 3:**
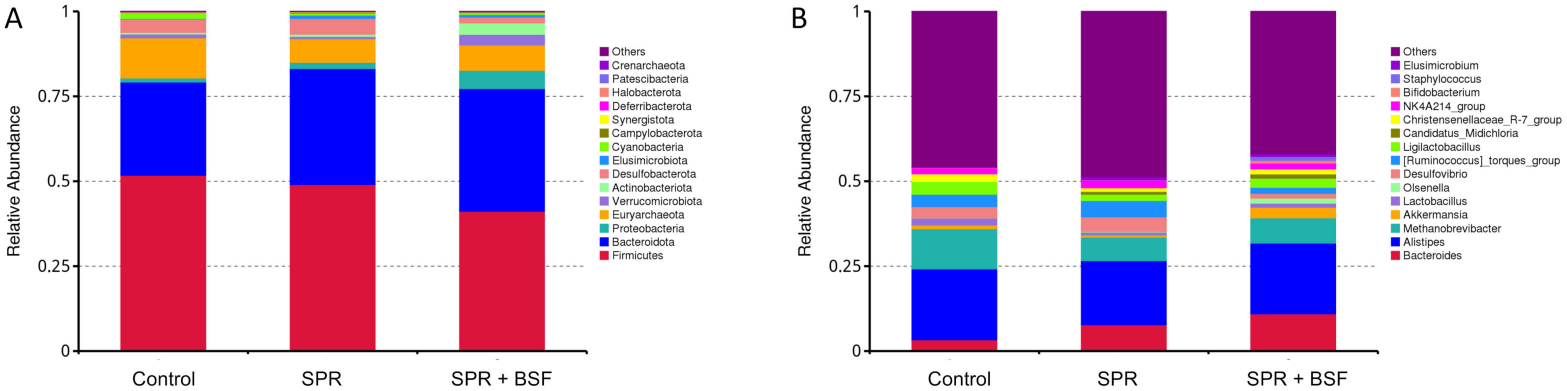
The microbial composition of cecal content representing the relative abundance at the phylum **(A)** and genus level **(B)** in chickens fed different diets. Diets: control: corn–soybean-based diet; SPR: the soybean in the control diet was partially replaced with local feedstuffs; SPR + BSF: black soldier fly dried larvae were added to the SPR diet.

The most abundant phyla and genera with high average relative abundance are presented in Figure 3. At the phylum level, Firmicutes and Bacteriodetes were the most dominant phyla in cecal samples. The relative abundance of Firmicutes and Bacteriodetes was 51.7 and 27.4% for chickens fed Control, 49.1 and 34.1% for chickens fed SPR, and 41.1 and 36.1% for chickens fed SPR+BSF diets, respectively (Figure 3A). At the genus level, *Bacteroides, Alistipes*, and *Methanobrevibacter* spp. had the highest relative abundance (Figure 3B).

**Figure 4:**
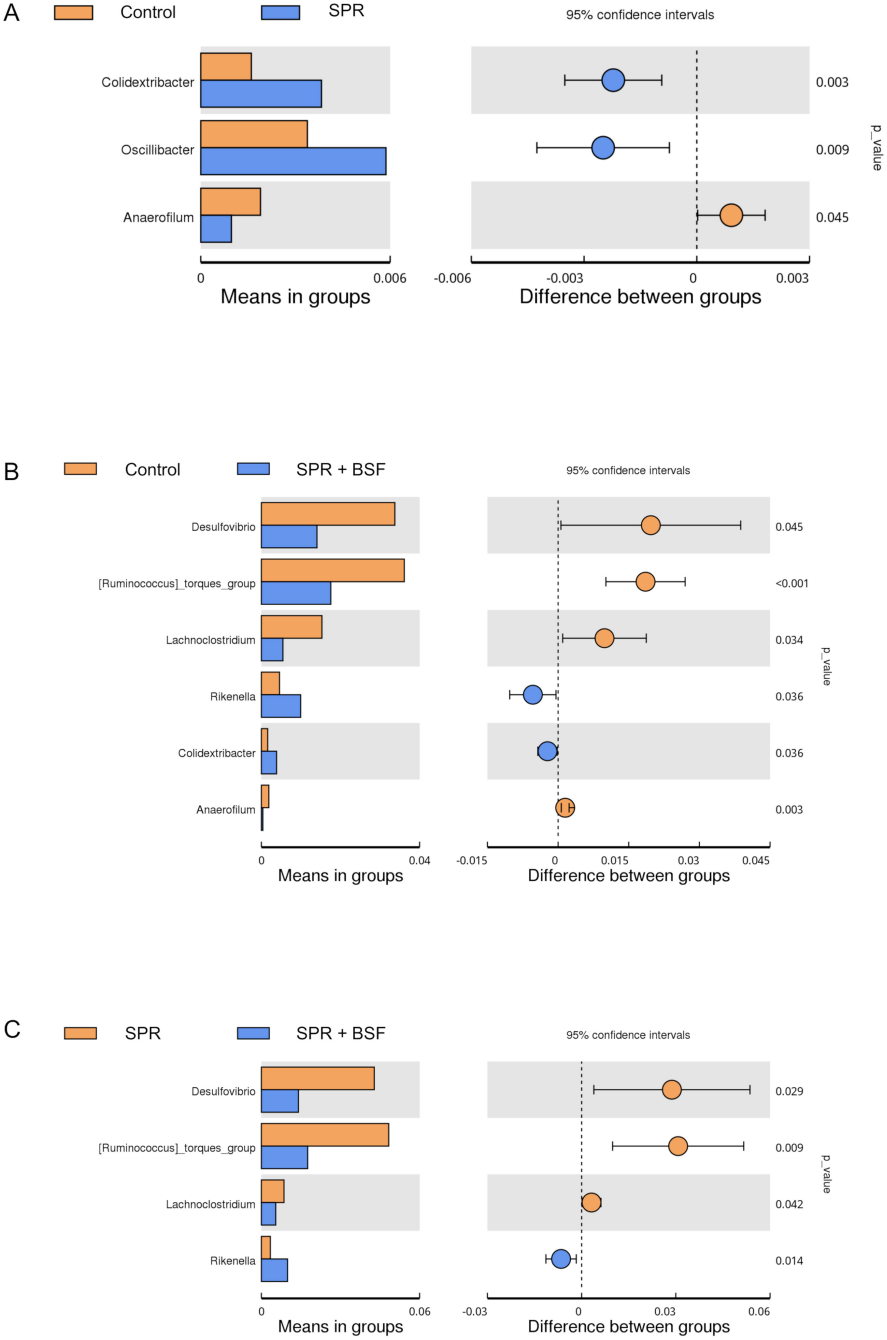
Comparisons of abundant bacteria at genus level in cecal samples of chickens: **(A)** control and SPR, **(B)** control and SPR + BSF, and **(C)** SPR and SPR + BSF diets (*P* < 0.05). Diets: control: corn–soybean-based diet; SPR: the soybean in the control diet was partially replaced with local feedstuffs; SPR + BSF: black soldier fly dried larvae were added to the SPR diet.

The bacteria composition of cecal samples at the genus level is presented in Figure 4. The abundance of *Colidextribacter, Oscillibacter*, and *Anaerofilum* spp. varied between chickens fed the Control and SPR diets. *Colidextribacter* and *Oscillibacter* spp. showed higher abundance in the ceca of chickens fed the SPR than those fed the control diet, while *Anaerofilum* spp. showed lower abundance in SPR compared to control (Figure 4A). *Desulfovibrio, Ruminococcus torques, Lachoclostridum*, and *Anaerofilum* spp. were significantly more abundant in the ceca of chickens fed the Control diet than those fed the SPR+BSF diet. In contrast, *Rikenella* and *Colidextribacter* spp. were more abundant in chickens fed SPR+BSF than those fed the Control diet (Figure 4B). When comparing cecal content from chickens from SPR and SPR+BSF, *Desulfovibrio, Ruminococcus torques*, and *Lachnoclostridium* spp. were significantly more abundant in chickens fed SPR than those fed SPR+BSF, and it was vice versa for *Rikenella* spp. (Figure 4C).

## Discussion

The current study was a part of a comprehensive project to investigate possibilities of reducing soybean use in chicken diets by replacing soybean with agri-industrial by-products with or without BSF larvae meal inclusion. Our previous study showed that SPR and SPR+BSF diets did not affect the performance of local and commercial broilers (4). As far as the authors' knowledge, this is the first study on the effect of BSF larvae meal inclusion into the diet together with agri-industrial by-products on the cecal microbial community of chickens, intestinal histomorphometry, and behavioral characteristics. Our results revealed significant alterations in histomorphometry of the digestive tract, and cecal microbiota through the dietary manipulations, but not in behavioral traits.

### Behavior

In this study, we did not find any significant effect of diet on behavioral traits examined. Our hypothesis was that the inclusion of agri-industrial by-products and their incorporation with BSF larvae meal could affect the behavior of chickens. The results did not confirm the study hypothesis. The crude fiber content of SPR and SPR+BSF diets was not far beyond acceptable levels for broilers and this may partly explain absence of diet effect on the behavior.

In earlier studies, the impact of the BSF larvae provision on the behavior of broilers was found to increase activity-related behavior when insect larvae were provided separately from the diets as an enrichment (12, 14). Ipema et al. (13) reported that scattering either dry or live larvae through the pen increased the activity of broilers compared to the control group, which did not include BSF. Increased locomotor activity could be associated with better leg health and welfare (47, 48), and low activity has been considered one of the possible causes of impaired leg health thus welfare (49). In our study, BSF was included in the diet as a larvae meal. Therefore, the absence of any impact of the SPR+BSF diet on behavioral traits would be expected. Indeed, Ipema et al. (13) reported that the time spent for drinking, walking-standing, resting, and foraging behavior of commercial broilers fed BSF larvae meal and oil incorporated diet were similar to those fed the control in accordance with our results. Overall, it is clear that SPR and SPR+BSF did not result in a negative effect on the behavior of broilers under the experimental conditions.

### Intestinal histomorphometry

Mainly, the VH and area are associated with high levels of digestible nutrients in the diet (50). Increases in the VH indicate an increased nutrient absorption area, which may allow better growth performance (20). Since, the growth rate of chickens fed the SPR and SPR+BSF diets was found to be similar to those fed the Control diet (4), we hypothesized that the SPR and SPR+BSF diets would not negatively affect the morphological characteristics of the intestine. Indeed, no previous study has investigated the effect of sunflower meal, brewers' dried grain, wheat middlings, and BSF meal in the same diet on intestinal histology.

Decreased VH and CD in duodenum and jejunum were reported in broilers fed at increasing levels, from 70 to 210 g/kg, of sunflower meal (51) and from 50 to 200 g/kg of sunflower cake (52). Brewers' dried grain inclusion higher than 120 g/kg reduced jejunal VH (53). There are conflicting results regarding the effects of BSF on the histomorphometry of the intestine, depending on the strain, the inclusion level, and processing method, e.g., BSF live larvae, BSF whole larvae meal, or defatted or oil. He et al. (54) reported that supplementing the diet of Xuefeng black-bone chickens with from 1 to 3% BSF larvae meal might benefit the intestinal histomorphometry while 5% could decrease VH and VH/CD of the jejunum. It has been reported that inclusion of 3, 6, and 9% of BSF larvae meal did not affect duodenum, jejunum, and ileum histomorphometry in laying-type chicks (55). Dabbou et al. (23) found no effect of a diet including 5% of BSF-defatted meal on VH, CD, and VH/CD ratio; in particular, 15% of BSF-defatted meal lowered the VH/CD ratio. However, the replacement of soybean oil with BSF oil reduced CD (56). Contrary to this finding, Schiavone et al. (57) found no effect of BSF fat inclusion on the VH and CD of broilers' duodenum, jejunum, and ileum.

In our study, while the SPR and SPR+BSF diets reduced the VH in the duodenum, the jejunum and ileum, VH of chickens fed the SPR+BSF diet was similar to those of chickens fed the Control diet. This result may indicate that cell mitosis activation in the jejunum and ileum of chickens fed the SPR+BSF diet was similar to those fed the Control diet. On the other hand, the SPR diet resulted in the highest villi in the ileum. This different effect of diets on VH may be due to the fiber type differences among the diets, since different fiber types were reported as a determining factor in intestinal development (58). A higher VH/CD ratio is associated with better nutrient absorption (59) and can be used to determine intestinal integrity and evaluate the bird's response to diets (60). Notably, the diets did not influence the VH/CD ratio. De Verdal et al. (61) reported that VH/CD ratio decreased from 7.73 to 4.94 from the duodenum to the ileum, similar to the VH/CD ratio obtained in the present study. Considering our previous results (4) showing that chickens' growth performance and feed efficiency were not influenced by SPR and SPR+BSF diets, we assume that absorption capacity was similar in all dietary groups.

### Microbiome composition of the cecum

The diet is one of the factor contributing the composition of the gut microbiota, including the ceca (62). It is assumed that replacing soybean with agri-industrial by-products and BSF larvae meal will positively affect the cecal microbiota of chickens, depending on the fiber and amino acid contents of the dietary composition. In the present study, we have examined the modifications in the cecal microbiota profile of chickens by utilizing 16S rRNA gene sequencing. The higher Chao1 index, which estimates total species richness, in chickens fed SPR + BSF than those on the control diet indicated a higher cecal microbiota richness in the SPR + BSF chickens, but the overall complexity of the microbial community was stable. Furthermore, PCoA analysis showed that partial substitution of soybean with SPR and SPR+BSF did not cause a significant compositional change in the cecal microbiota; therefore, the microbiota constitution of the samples did not reveal any evident clustering. This result could potentially arise from the shared common environment in which the chickens were raised. Additionally, the diets, although differing in content, may have offered similar overall nutritional profiles and microbial substrates. The cecal microbiota may also have a robust core microbiome that sustains stability despite dietary changes, especially when dietary changes are not significant. Furthermore, it is possible that the chickens were at a developmental stage where their microbiota had already stabilized, and that the depth of sequencing and sampling was insufficient to identify minute variations in the microbiota composition.

Firmicutes, Bacteroidetes (also known as Bacteroidota), and Proteobacteria constituted the predominant phyla, making up nearly 80% of all bacterial populations across all dietary groups. This finding aligns with findings from previous research (30, 63, 64). Furthermore, *Bacteroides, Alistipes, Methanobrevibacter, Akkermansia*, and *Lactobacillus* spp. emerged as the first five most prevalent genera in all samples, consistent with observations in similar studies (30, 63, 64). *Bacteroides* and *Alistipes* spp. in the cecum are related to dietary fiber fermentation, producing acetic acid, and are considered beneficial bacteria for the gastrointestinal system. *Methanobrevibacter* spp. correlates with avian performance-related outcomes (65). Metabolic activation of *Lactobacill*us may improve intestinal health by lowering pH and thus play a role against pathogenic infection (66).

Using the agar plate technique, Yaqoob et al. (33) showed that partial replacement of soybean up to 9% of sunflower meal increased cecal-beneficial bacteria, such as *Lactobacillus* and *Bifidobacterium* spp., while there was no effect of dietary treatments on cecal microbial counts. It was reported that a diet containing 25% sunflower meal decreased Ruminococcaceae and Lachnospiraceae in chickens (67). In laying ducks up to 20% sunflower meal replacement reduced Spirochaetes, which can cause enteric disease (68). Brewers' yeast increased *Bacillus* and *Enterococcus* spp. in excreta in broilers (69). In the present study, 16S rRNA gene sequencing has revealed that chickens fed the SPR diet showed notably higher levels of *Colidextribacter* and *Oscillibacter* spp. compared to those on the control diet. The increased abundance of these bacteria has been associated with elevated SCFA levels and decreased TNF-α levels, which indicate improved gut health (70). The increased SCFAs producing bacteria in the cecal content of the chickens fed the SPR diet could be related to higher fiber content. In addition, both *Colidextribacter* and *Oscillibacter* spp. were shown to be associated with healthy liver (71). *Colidextribacter* spp. can also promote inosine production, which helps to regulate inflammatory responses and maintain the integrity of the intestinal mucosa (72–74). In light of these findings, it could be concluded that the SPR diet positively regulated cecal microbiota. The higher abundance of *Anaerofilum* spp. in chickens fed the Control diet compared to the chickens fed the SPR diet may be related to the higher percentage of abdominal fat (75); however, abdominal fat weight was not measured in the present study.

The inclusion of BSF larvae meal or oil in chicken diets has been shown to affect the cecal microbiota of chickens in many studies (34, 76). However, in most studies, the BSF effect on microbiota depends on the inclusion level and BSF feeding duration/period. It was shown that 5% of BSF meal inclusion positively influenced the cecal microbiota, increasing beneficial bacteria; however, 15% of BSF may have a negative influence on microbial complexity (77). de Souza Vilela et al. (36) reported that 20% BSF meal inclusion in the finisher diets of broilers had a minor effect on microbiota in caeca. In our study, BSF larvae meal was included in the SPR diet from the day of the hatch to the slaughter age. Compared to SPR+BSF, the control diet significantly increased the abundance of *Ruminococcus_torques_group* and L*achnoclostridium* spp., which were associated with short-chain fatty acid-producing bacteria (28), and chickens' growth performance (78), and the abundance of *Desulfovibrio* spp., which contributes to the cleansing of free hydrogen formed during anaerobic fermentation (30). In SPR+BSF-fed chickens, the abundance of *Colidextribacter* and *Rikenella* spp. was higher than in those fed the Control diet. The higher abundance of *Rikenella* spp. in chickens fed with the SPR+BSF diet could be associated with the improvement of the intestinal flora environment and might alleviate intestinal inflammation (79). Similar changes were obtained for *Colidextribacter* and *Anaerofilum* spp. in the ceca of chickens fed SPR and SPR+BSF diets indicated that these changes were mainly based on the SPR diet.

## Conclusion

In conclusion, the results of the microbiome profile suggested that the SPR diet was associated with increased abundance of *Oscillibacter* and *Colidextribacter* spp. in the ceca. The BSF inclusion into the SPR diet could further improve the intestinal flora by increasing the abundance of *Rikenella* spp. Although some variations were observed in intestinal histomorphometry, similar villi-to-crypt ratios obtained in chickens fed control and experimental diets indicated no significant alterations in the absorptive capacity of the digestive system among the dietary groups. SPR and SPR+BSF diets did not result in any negative effect on the behavior of broiler chickens under the experimental conditions. Further research would examine the impact of each by-product separately and possible interactions among them to expand our understanding.

## Data Availability

The datasets presented in this study can be found in online repositories. The names of the repository/repositories and accession number(s) can be found at: https://www.ncbi.nlm.nih.gov/bioproject/1134447, PRJNA1134447.
